# Complex cytokine profiles induced by BCG vaccination in UK infants

**DOI:** 10.1016/j.vaccine.2009.11.004

**Published:** 2010-02-10

**Authors:** Maeve K. Lalor, Steven G. Smith, Sian Floyd, Patricia Gorak-Stolinska, Rosemary E. Weir, Rose Blitz, Keith Branson, Paul E. Fine, Hazel M. Dockrell

**Affiliations:** a Immunology Unit, Department of Infectious and Tropical Diseases, London School of Hygiene & Tropical Medicine, Keppel Street, London WC1E 7HT, UK; b Infectious Disease Epidemiology Unit, Department of Epidemiology and Public Health, London School of Hygiene & Tropical Medicine, Keppel Street, London WC1E 7HT, UK

**Keywords:** BCG vaccination, Infant immune response, Cytokines

## Abstract

IFNγ plays an important part in immunity to tuberculosis (TB), but although it is necessary, it is not on its own sufficient for protection against TB. To identify other cytokines that play a role in the protection against TB induced by BCG vaccination, immune responses were compared between vaccinated and unvaccinated infants from the UK where BCG is known to provide protection. Twenty-one cytokines and chemokines were tested in supernatants from diluted whole blood cultures that had been stimulated for 6 days with *Mycobacterium tuberculosis* PPD. For 15 out of 21 of the cytokines tested responses were much higher in BCG vaccinated infants than in unvaccinated infants. These included: pro-inflammatory cytokines; IFNγ (median 1705 pg/ml vs. 1.6 pg/ml in vaccinated and unvaccinated infants, respectively), TNFα (median 226 pg/ml vs. 18 pg/ml), as well as IL-2, IL-1α and IL-6; TH2 cytokines: IL-4, IL-5 and IL-13 (median 104 pg/ml vs. 1.6 pg/ml); the regulatory cytokine IL-10 (median response 96 pg/ml vs. 8 pg/ml); the TH17 cytokine IL-17, chemokines (IP-10, MIP-1α and IL-8) and growth factors (GM-CSF and G-CSF). The greatest increase in cytokine production in BCG vaccinees compared to unvaccinated infants was seen with IFNγ. While responses for many cytokines were correlated with the IFNγ response, others including IL-17 and IL-10 were not. The pattern of cytokine induction following BCG vaccination is complex and measurement of one of two cytokines does not reveal the whole picture of vaccine-induced protection.

## 1 Introduction

Bacille Calmette-Guerin (BCG), the vaccine for protection against tuberculosis (TB), is currently given to most of the world's infants as part of the WHO's Expanded Program on Immunisation (EPI) [1]. Clinical trials of BCG show variable efficacy (0–80%) against pulmonary tuberculosis in adults [2], but high efficacy in infants against the severe forms of childhood tuberculosis [3].

Several new TB vaccines are being tested or are soon to be tested in clinical trials [4]. Some of these would be given as booster vaccines following BCG vaccination, and others are genetically modified BCG vaccines. Biomarkers of protection are urgently required to help assess these new TB vaccines, as without them clinical trials will be lengthy and require very large numbers of study subjects [5]. Studying immune responses to BCG vaccination in the UK, where BCG vaccination has been shown to provide 75% protection, gives us an opportunity to identify biomarkers of protection following successful vaccination against TB.

BCG vaccinated infants in the UK have been found to make an IFNγ response to M.tb PPD in stimulated 6-day whole blood cultures, while unvaccinated infants do not make a detectable IFNγ response [6]. Though the TH1 cytokine IFNγ plays an important part in immunity to TB [7–9], it is not sufficient on its own to protect against TB, and other cytokines, such as TNFα, also play a role in immunity to TB [5].

This study was designed to identify which cytokines other than IFNγ are induced following BCG vaccination in UK infants, and the associations between the various cytokines produced. The Multiplex assay has the advantage of being more sensitive than ELISA, and to be able to measure multiple cytokines in a small blood sample, and so is appropriate for studies of infants. The study aims to characterise cytokine patterns induced following vaccination against tuberculosis, which could, in turn, suggest promising candidates for biomarkers of protection for clinical trials of new TB vaccines.

## 2 Methods

### 2.1 Recruitment and study design

Twenty-eight Caucasian infants who were born in the UK, and who were part of our BCG vaccination study in which we had measured IFNγ in supernatants 3 months post-BCG vaccination by ELISA [6] were selected for additional cytokine analysis. Of these infants, 19 had been BCG vaccinated between 5 and 10 weeks of age (mean 7 weeks), and 9 had not received BCG. Approval for the study was given by the Redbridge and Waltham Forest Health Authority Local Research Ethics Committee, and the Ethics Committee of the London School of Hygiene & Tropical Medicine.

### 2.2 Whole blood assay, IFNγ ELISA

Whole blood assays and ELISAs for IFNγ were carried out as previously described [10,11]. Heparinised whole blood was diluted 1 in 10 and cultured on the day of collection with the M.tb PPD (Statens Serum Institut, Copenhagen (SSI), RT49, lot 204) at a concentration of 5 μg/ml or medium alone (unstimulated) as the negative control. PHA-P was used as a positive control; IFNγ from PHA-P cultures was measured by ELISA [6] but were not included in the Multiplex assay. Cultures were incubated at 37 °C with 5% CO_2_; supernatants were harvested on day 6 and stored at −70 °C until assayed for IFNγ in single 100 μl samples by quantitative ELISA or for 21 cytokines and chemokines in single 25 μl samples by Multiplex assay.

### 2.3 21-plex Multiplex

The following 21 cytokines and chemokines were measured simultaneously in supernatants using a human cytokine Lincoplex premixed kit according to the manufacturer's instructions (cat #HCYTO-60K-PMX, Linco Research Inc., St. Charles Missouri, USA): IL-1β, IL-2, IL-4, IL-5, IL-6, IL-7, IL-8, IL-10, IL-12p70, IL-13, IL-15, IL-17, IL-1α, IFNγ, G-CSF, GM-CSF, TNFα, Eotaxin, MCP-1, MIP-1α and IP-10. Unstimulated, M.tb PPD stimulated and 1 in 10 diluted M.tb PPD stimulated samples were read on the Biorad Luminex reader using Bioplex manager 4.1 software. For each cytokine the standard curve ran from 3.2 to 10,000 pg/ml. The reproducibility of the assay for individual cytokines and chemokines was determined using the quality controls provided with the kit. For all 21 cytokines and chemokines, the coefficients of variation for the low control were 7.5% or less. There was greater variation in the high control: 15 cytokines had coefficients of variation below 25%, but for 6 cytokines the variation was greater (26–44%). However, as only 8/588 data values presented were within the high range of these cytokines we believe this variation will have had only a small effect on the data presented.

### 2.4 Statistical analysis

Data were analysed using Stata 10. Unstimulated cytokine responses were subtracted from antigen stimulated results. For Multiplex, data values below 3.2 pg/ml were assigned as 1.6 pg/ml and for values over the detection limit the 1/10 diluted sample result was multiplied by 10 and used. For MCP-1, IL-8 and IP-10, some values were above the detection limit and were assigned 30,000 pg/ml for MCP-1 and IP-10, and 100,000 pg/ml for IL-8, assessed by looking at the highest values that were measured for those chemokines. One TNFα measurement was excluded as the unstimulated sample had higher levels of TNFα than the M.tb PPD stimulated sample.

Non-parametric Mann–Whitney tests were used to compare cytokine responses between vaccinated and unvaccinated infants. Median fold differences were calculated, and correlations between IFNγ measured by ELISA or Multiplex, and between different cytokines measured by Multiplex, were assessed by calculating a Spearman's rank correlation.

Principal components analysis was conducted on the log cytokine data from vaccinated infants (*n* = 18), restricted to fifteen cytokines (IL-1α, IL-2, IL-6, TNFα, IFNγ, IL-17, IL-4, IL-5, IL-13, IL-10, IL-8, IP-10, MIP-1α, G-CSF and GM-CSF) for which there was evidence of a difference between unvaccinated and vaccinated infants (*P* < 0.01). (One infant was excluded as their TNFα value was not included in the analysis.) The principal components analysis was done on “standardised” log cytokine measurements (with the mean response subtracted from the observed value, and this value divided by the standard deviation), by using the correlation matrix for the identification of principal components. Principal components analysis was then conducted restricted to particular groups of cytokines; pro-inflammatory cytokines (IL-1α, IL-2, IL-6, TNFα and IFNγ), and TH2 cytokines (IL-4, IL-5, IL-13).

## 3 Results

**Fig. 1:**
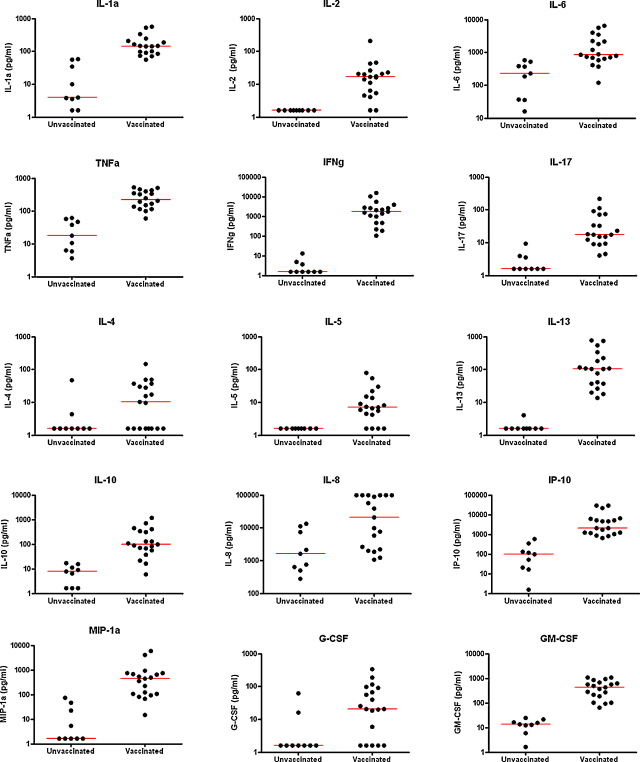
Cytokine responses in BCG vaccinated and unvaccinated infants. Cytokines in supernatants from diluted whole blood cultures which were stimulated with M.tb PPD for 6 days were measured using a Multiplex assay in infants who had been BCG vaccinated 3 months previously and in unvaccinated control infants. Pro-inflammatory cytokines IL-1α, IL-2, IL-6, TNFα and IFNγ, TH17 cytokine IL-17, TH2 cytokines IL-4, IL-5, IL-13, chemokines IL-8, IP-10, MIP-1α and growth factors G-CSF, GM-CSF.

Of the vaccinated infants, 4/19 made relatively low (<500 pg/ml) IFNγ responses, 8/19 made high (>500 pg/ml, <2000 pg/ml) IFNγ responses, and 7/19 made very high IFNγ responses (>2000 pg/ml) in cultures stimulated with M.tb PPD, as measured by ELISA. IFNγ to M.tb PPD measured by Multiplex correlated very strongly with the IFNγ measured in the ELISA (*r* = 0.9). For 15 of the 21 cytokines tested there was strong evidence that responses in the vaccinated infants were higher than in the unvaccinated infants (Table 1, Fig. 1). There was no or weak associations between cytokine responses and lymphocyte numbers (data not shown).

For the purposes of presenting the results from so many cytokines, cytokines have been grouped into pro-inflammatory cytokines (IFNγ, IL-2, TNFα, IL-1α and IL-6), TH2 cytokines (IL-4, IL-5, IL-13), regulatory cytokine (IL-10), TH17 cytokine (IL-17), chemokines (IL-8, IP-10, MIP-1α) and growth factors (G-CSF and GM-CSF), although we recognise that these groupings may be over-simplistic.

Five pro-inflammatory cytokines were strongly induced by BCG vaccination: IFNγ (*P* < 0.0001) which had a median value of 1705 pg/ml in the vaccinated group compared with 1.6 pg/ml in the unvaccinated group, TNFα (226 pg/ml vaccinated vs. 18 pg/ml unvaccinated, *P* < 0.0001), IL-2 (17 pg/ml vaccinated vs. 1.6 pg/ml unvaccinated, *P* < 0.0001), IL-1α (145 pg/ml vaccinated vs. 4 pg/ml unvaccinated, *P* < 0.0001) and IL-6 (855 pg/ml vaccinated vs. 227 pg/ml unvaccinated, *P* = 0.0003). There was also strong evidence that the pro-inflammatory cytokine IL-17 was induced by BCG vaccination (17 pg/ml vaccinated vs. 1.6 pg/ml unvaccinated, *P* < 0.0001).

There was strong evidence that three TH2 cytokines were also induced by BCG vaccination: IL-4 (10 pg/ml vaccinated vs. 1.6 pg/ml unvaccinated, *P* = 0.013), IL-5 (7 pg/ml vaccinated vs. 1.6 pg/ml unvaccinated, *P* = 0.0005) and IL-13 (104 pg/ml vaccinated vs. 1.6 pg/ml unvaccinated, *P* < 0.0001).

There was also strong evidence that the regulatory cytokine IL-10 was induced by BCG vaccination (96 pg/ml vaccinated vs. 8 pg/ml unvaccinated, *P* < 0.0001). Three chemokines: IL-8 (20,562 pg/ml vaccinated vs. 1621 pg/ml unvaccinated, *P* = 0.0073), IP-10 (2122 pg/ml vaccinated vs. 99 pg/ml unvaccinated, *P* < 0.0001) and MIP-1α (454 pg/ml vaccinated vs. 1.6 pg/ml unvaccinated, *P* < 0.0001) were induced by BCG vaccination. The growth factors G-CSF (21 pg/ml vaccinated vs. 1.6 pg/ml unvaccinated, *P* = 0.012) and GM-CSF (420 pg/ml vaccinated vs. 14 pg/ml unvaccinated, *P* < 0.0001) were also induced. There were six cytokines (IL-1β, IL-7, IL-12p70, IL-15, Eotaxin and MCP-1) for which there was no statistical evidence of a median difference between responses in vaccinated and unvaccinated infants, and (with the exception of Eotaxin) the median responses were either very similar in the two groups or higher in the unvaccinated group (Table 1).

**Fig. 2:**
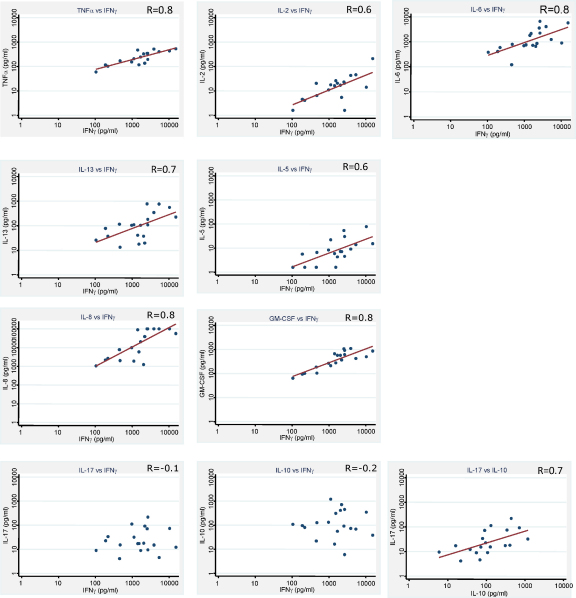
Cytokine correlations in BCG vaccinated. Cytokines were measured using a Multiplex assay in supernatants from diluted whole blood cultures which were stimulated with M.tb PPD for 6 days in infants who had been BCG vaccinated 3 months previously.

Correlations between cytokines where there was evidence of a difference between vaccinated and unvaccinated infants were examined by Spearman's rank correlation, among the vaccinated group (Table 2). Eight out of 14 cytokines correlated moderately strongly or strongly with IFNγ, and ten correlated with TNFα. IFNγ and TNFα correlated strongly with each other (*r* = 0.8). IFNγ and TNFα correlated with pro-inflammatory cytokines such as IL-2 with IFNγ (*r* = 0.6) and IL-2 with TNFα (*r* = 0.6) and IL-6 with IFNγ (*r* = 0.8), but also with TH2 cytokines such as IL-13 with IFNγ (*r* = 0.7) and IL-5 with IFNγ (*r* = 0.6). IFNγ and TNFα also correlated with chemokines and growth factors, for example IFNγ with IL-8 (*r* = 0.8) and IFNγ with GM-CSF (*r* = 0.8) (Fig. 2).

Cytokines for which there was no statistical evidence of correlation with IFNγ or TNFα included IL-17 and IL-10. IL-17 and IL-10 were correlated with each other (*r* = 0.7, Fig. 2), however the correlations between IL-10 or IL-17 and other cytokines, were weak and negative (Fig. 2). Adding the “standardised” TH1 responses together (IFNγ, TNFα, IL-1α, IL-6 and IL-2), and calculating the correlation with the “standardised” IL-10 response, gave a correlation coefficient of −0.4, which was considerably larger in magnitude than any of the individual correlations between a TH1 cytokine and IL-10.

**Fig. 3:**
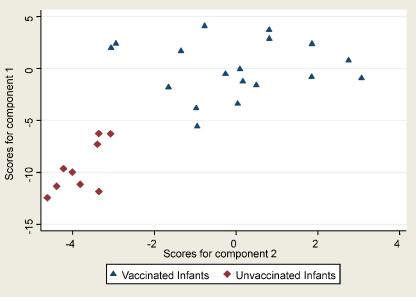
Score values for components 1 and 2. Score values for vaccinated (triangles) and unvaccinated infants (diamonds) for components 1 and 2 from the principal components analysis.

From the principal components analysis, 90% of the total variation in the responses of the 15 cytokines could be summarised by 5 components. The first component alone accounted for 49% of the total variation and corresponded approximately to the average of the “standardised” log responses to IFNγ, IL-1α, IL-2, IL-6, TNFα, IL-5, IL-13, IL-8, MIP-1α, G-CSF and GM-CSF. The second component is independent of the first one, and describes a further 20% of the remaining variation and corresponded approximately to the average of the “standardised” log response to IL-4, IL-5, IL-10, IL-17 and IP-10 (Table 3). Using the two components to explain the variation within the 15 cytokines included, the vaccinated and unvaccinated infants were clearly separated into two groups and also the variation among individuals who were vaccinated was much more simply summarised (Fig. 3). Principal component analysis of the five pro-inflammatory cytokines measured showed that 73% of the total variation could be explained by the first component, and this corresponded approximately to the average “standardised” response to the 5 cytokines.

## 4 Discussion

We have previously shown that BCG vaccinated infants in the UK made IFNγ to M.tb PPD in 6-day diluted whole blood cultures, while unvaccinated infants did not make a detectable IFNγ response [6].

The Multiplex assay enabled us to test for multiple cytokines in the same supernatant sample, and 6 out of the 21 cytokine responses tested showed no evidence of a difference in production between the vaccinated and unvaccinated infants. These included IL-12p70, IL-1β, IL-15, Eotaxin, and IL-7 which were present in very low to undetectable concentrations in supernatants of stimulated cultures for both vaccinated and unvaccinated infants. This may be due to the cytokines not being produced in M.tb PPD stimulated cultures during the 6 days of culture at this time point since vaccination, i.e. at 3 months post-BCG vaccination, to their being produced but not remaining in the supernatant for the 6 days of culture, or to their being produced at levels undetectable by the Multiplex assay despite the increased sensitivity of this assay compared to ELISA. Responses to MCP-1 were seen in both vaccinated and unvaccinated infants and may reflect non-mycobacterial specific responses. We showed that BCG vaccination induced pro-inflammatory cytokines such as IFNγ and TNFα which are known to activate M.tb infected macrophages, and IL-2 which promotes stimulation of TH1 cells and CD8 T cells. We also showed that BCG vaccination induced IL-1α and IL-6 following BCG vaccination. There is little known about the role of IL-1α in immunity to TB; a TB case–control study in the Gambia suggested it may play a role in TB susceptibility [12]. In TB patients from Pakistan IL-6 was shown to be increased in Culture Filtrate Protein stimulated supernatants compared to controls [13], and in South African TB patients IL-6 was increased in plasma compared to healthy endemic controls [14]. IL-6 has been regarded as a pro-inflammatory cytokine, however it has been shown to display anti-inflammatory properties which can inhibit TNFα production in CD8 T cell supernatants stimulated with mycobacterial fractions [15].

We were interested in whether those infants with greater IFNγ responses also made greater pro-inflammatory cytokine responses and smaller TH2 cytokine responses. We found that IFNγ responses correlated positively with production of 9 cytokines including the other pro-inflammatory cytokines measured, but also with that of the TH2 cytokines IL-5 and IL-13 and with the chemokine IL-8 and growth factor GM-CSF. The greatest fold difference between vaccinated and unvaccinated cytokine responses was seen for IFNγ. This, along with the strong evidence for correlations with many different types of cytokine, highlights the importance of IFNγ in immunity for TB induced by BCG vaccination.

Interestingly, IL-17 (a pro-inflammatory cytokine produced by the recently described TH17 T cell subset [16]) was induced by BCG vaccination, but there was no evidence that it correlated with the IFNγ response. This may imply that, if there is TH17 mediated immunity induced by BCG vaccination, it is independent of the IFNγ mediated immunity and may be produced by different cells than those which produce IFNγ. IL-17 has been shown to play a role in autoimmune disease [17–19], but has also recently been thought to play a role in M.tb infection [20], as it was shown to upregulate chemokines which led to increased recruitment of TH1 cells [21], and is also thought to recruit neutrophils to facilitate granuloma formation [22]. There is evidence that TB patients produce less IL-17 following overnight culture with ESAT6/CFP10 than contacts [23]. IL-17 has also been shown to regulate IFNγ production in cell cultures stimulated with M.tb in TB patients [24], and the IL-17 producing CD4+ T cells had characteristics of long lived central memory cells but many do not produce IFNγ [25].

The role of TH2 cytokines such as IL-4, IL-5 and IL-13 in the immune response to *Mycobacterium tuberculosis* has been debated, and it has been suggested that TH2 responses reflect inappropriate or suboptimal immune responses to mycobacteria [26]. Several human studies have shown that IL-4 production is increased in tuberculosis patients compared with controls [27–30]. Studies in mice with disrupted IL-4 genes showed there was no evidence of a change in resistance to M.tb infection [31], although with respect to IL-4 some mouse models do not provide a good model of human immunopathology [32]. It is possible that the TH2 cytokine responses and the IL-10 responses do not simply reflect a regulation of the IFNγ responses, but may also reflect that there is a polyclonal response of mixed T cell populations, and some of the IL-10 measured may be produced by fully differentiated TH1 T cells [33,34].

In Malawian infants, a smaller increase in TH1 cytokines has been seen following BCG vaccination than in the UK [6], and one hypothesis for this is that there may be suppression/immunoregulation by TH2 cytokines and/or by T regulatory cells and IL-10. We found a significant increase in TH2 cytokines IL-4, IL-5 and IL-13, and also in the regulatory cytokine IL-10 following BCG vaccination in UK infants who we presume made an immune response to BCG that was protective against the disseminated childhood forms of TB. The high levels of TH2 cytokines seen in the UK vaccinated infants may have been produced in response to the high levels of IFNγ produced, in order to regulate the IFNγ response. IL-5 and IL-13 both correlated positively with the IFNγ response in vaccinated infants, but the correlation between the IL-10 and IFNγ response was weak and negative. There was stronger evidence of a negative association between pro-inflammatory responses and IL-10 when all pro-inflammatory responses were added together, possibly suggesting that IL-10 regulates the entire pro-inflammatory cytokine profile.

Chemokines have been shown to be important in immunity to tuberculosis [35], particularly in cellular trafficking for granuloma formation [36]. We found that the chemokines IL-8 (CXCL8), IP-10 (CXCL10) and MIP-1α (CCL3) were all induced by BCG vaccination.

The growth factors G-CSF and GM-CSF were also increased in BCG vaccinated infants; GM-CSF has been shown to have many roles in immunity to TB such as inducing the generation and proliferation of cells such as macrophages, DCs and neutrophils, but also by acting to recruit leukocytes and to enhance APC function and may be necessary for optimum T cell immunity [37,38].

Principal components analysis was performed in order to reduce the dimensionality of the data, to attempt to summarise the overall pattern of response among the 15 cytokines. We summarised 68% of the total variation in the data by using just 2 components. These two components suggest that all 15 cytokines and chemokines measured are important, rather than just a particular subset, and that all 15 cytokines and chemokines are useful in describing the variation in immune response among individuals.

This study identified several cytokines and chemokines (IFNγ, TNFα, IL-2, IL-6, IL-1α, IL-4, IL-5, IL-13, IL-10, IL-8, IP-10, MIP-1α, G-CSF, GM-CSF) which when measured together could be used as biomarkers for protection in future studies and clinical trials. Further studies examining cytokine responses in individuals from populations in which BCG does not offer good protection have been planned, and studies to establish which cells are producing these cytokines, and the kinetics involved, are warranted.

Polyfunctional CD4+ T cells have recently been shown to be induced following BCG and recombinant MVA85A vaccination [39]. We suggest that future vaccine trials might measure cytokines released into supernatants by Multiplex as a first step, in order to identify key cytokines for more detailed study, followed by measurement of these key cytokines and chemokines using multicolour FACS to determine if polyfunctional cells have been induced. With the current focus on polyfunctional cells [25,39,40], this study reminds us of the importance of measuring additional cytokines and chemokines to assess vaccine-induced immunity, and not just to focus on those we know are important.

## Figures and Tables

**Table 1:** Median response in 21 cytokines and chemokines.

	Cytokine	Unvac median (pg/ml)	Vac median (pg/ml)	Median fold difference	*P* value
Pro-inflammatory cytokine	IFNγ	1.6	1705	1066	*P* < 0.0001
	TNFα	18	226	13	*P* < 0.0001
	IL-2	1.6	17	11	*P* < 0.0001
	IL-1α	4	145	36	*P* < 0.0001
	IL-6	227	855	4	*P* = 0.0003

TH17 cytokine	IL-17	1.6	17	11	*P* < 0.0001

TH2 cytokine	IL-4	1.6	10	6	*P* = 0.013
	IL-5	1.6	7	4	*P* = 0.0005
	IL-13	1.6	104	65	*P* < 0.0001

Regulatory cytokine	IL-10	8	96	12	*P* < 0.0001

Chemokine	IL-8	1621	20,562	13	*P* = 0.0073
	IP-10	99	2122	21	*P* < 0.0001
	MIP-1α	1.6	454	284	*P* < 0.0001

Growth factor	GCS-F	1.6	21	13	*P* = 0.012
	GM-CSF	14	420	30	*P* < 0.0001

No evidence of a difference between vaccinated and unvaccinated infants	IL12p70	1.6	1.6	1	*P* = 0.49
	IL-15	1.6	1.6	1	na
	Eotaxin	1.6	7	4	*P* = 0.13
	MCP-1	6932	1560	0	*P* = 0.54
	IL-1β	1.6	1.6	1	*P* = 0.08
	IL-7	1.6	1.6	1	*P* = 0.12

Supernatants from diluted blood samples stimulated with M.tb PPD for 6 days were tested for 21 cytokines and chemokines from unvaccinated (Unvac) and vaccinated (Vac) infants 3 months post-BCG vaccination. Median fold differences and *P* values for significance testing by the Mann–Whitney test are also presented (na is not applicable).

**Table 2:** Spearman's correlation coefficients (*r*) of cytokine responses.

	IFNγ	TNFα	IL-2	IL-1α	IL-6	IL-17	IL-4	IL-5	IL-13	IL-10	IL-8	IP-10	MIP-1α	G-CSF	GM-CSF
IFNγ	1.0														
TNFα	0.8	1.0													
IL-2	0.6	0.6	1.0												
IL-1α	0.6	0.7	0.4	1.0											
IL-6	0.8	0.7	0.6	0.7	1.0										
IL-17	−0.1	−0.1	−0.5	0.1	−0.1	1.0									
IL-4	0.3	0.2	0.2	0.0	0.2	0.3	1.0								
IL-5	0.6	0.7	0.2	0.5	0.4	0.4	0.4	1.0							
IL-13	0.7	0.7	0.5	0.3	0.5	0.0	0.2	0.7	1.0						
IL-10	−0.2	−0.3	−0.4	−0.2	−0.3	0.7	0.5	0.3	−0.2	1.0					
IL-8	0.8	0.7	0.4	0.4	0.7	0.0	0.1	0.5	0.7	−0.3	1.0				
IP-10	0.4	0.2	0.3	0.0	0.3	0.3	0.6	0.4	0.3	0.4	0.2	1.0			
MIP-1α	0.5	0.7	0.4	0.8	0.7	−0.2	−0.2	0.4	0.4	−0.5	0.6	−0.2	1.0		
G-CSF	0.4	0.6	0.4	0.9	0.5	0.0	−0.2	0.4	0.2	−0.3	0.3	−0.1	0.8	1.0	
GM-CSF	0.8	0.8	0.6	0.7	0.8	−0.1	0.2	0.4	0.6	−0.4	0.7	0.3	0.8	0.6	1.0

Spearman's correlation coefficients (*r*) are presented from supernatants from diluted blood samples stimulated with M.tb PPD for 6 days from BCG vaccinated infants 3 months post-vaccination. Shaded boxes show *r* values 0.6 and above.

**Table 3:** Principal components analysis.

Component	Eigenvalue	Difference	Proportion	Cumulative
Component 1	7.28	4.3	0.49	0.49
Component 2	2.98	1.64	0.2	0.68
Component 3	1.33	0.33	0.09	0.77
Component 4	1.01	0.18	0.07	0.84
Component 5	0.83	0.35	0.06	0.9
Component 6	0.48	0.15	0.03	0.93
Component 7	0.33	0.09	0.02	0.95
Component 8	0.24	0.08	0.02	0.97
Component 9	0.16	0.05	0.01	0.98
Component 10	0.11	0.02	0.01	0.98
Component 11	0.08	0.02	0.01	0.99
Component 12	0.07	0.03	0	0.99
Component 13	0.04	0.01	0	1
Component 14	0.03	0.03	0	1
Component 15	0.01		0	1

Variable	Component 1	Component 2
Log IFNγ	0.33	0.11
Log IL-2	0.26	−0.1
Log IL-4	0.08	0.45
Log IL-5	0.25	0.3
Log IL-6	0.3	−0.09
Log IL-8	0.31	−0.01
Log IL-10	−0.11	0.5
Log IL-13	0.25	0.13
Log IL-17	0.01	0.39
Log IL-1α	0.31	−0.15
Log G-CSF	0.26	−0.16
Log GM-CSF	0.34	0.03
Log TNFα	0.34	0.06
Log MIP-1α	0.3	−0.17
Log IP-10	0.1	0.43

Principal components analysis of 15 cytokines from supernatants stimulated with M.tb PPD from infants 3 months post-BCG vaccination. Shaded boxes show important cytokines in the component.
